# Decorrelation Signal of Diabetic Hyperreflective Foci on Optical Coherence Tomography Angiography

**DOI:** 10.1038/s41598-018-27192-9

**Published:** 2018-06-11

**Authors:** Tomoaki Murakami, Kiyoshi Suzuma, Yoko Dodo, Tatsuya Yoshitake, Shota Yasukura, Hideo Nakanishi, Masahiro Fujimoto, Maho Oishi, Akitaka Tsujikawa

**Affiliations:** Department of Ophthalmology and Visual Sciences, Kyoto University Graduate School of Medicine, Kyoto, Japan

## Abstract

Diabetic hyperreflective foci in the outer retinal layers are a clinically relevant finding on optical coherence tomography (OCT) images, although their characteristics remain to be elucidated. Here we investigated the decorrelation signal around hyperreflective foci on OCT angiography (OCTA) images in diabetic retinopathy (DR). We retrospectively reviewed sufficient quality OCTA images from 102 eyes of 66 patients that were obtained using split-spectrum amplitude-decorrelation angiography algorithm. Most confluent hyperreflective foci were randomly deposited or appeared in a radiating array on the en-face structural OCT images in the inner nuclear layer (INL) or Henle’s fiber layer (HFL), respectively. Within the INL, hyperreflective foci were not accompanied by decorrelation signals and attached to capillaries on OCTA images. Decorrelation signals were sometimes delineated in hyperreflective foci in the HFL and other times appeared to be pseudopod-like or wrapping around hyperreflective foci, referred to as *reflectance-decorrelated foci*. The decorrelation signal intensity of hyperreflective foci in the HFL was associated with logMAR VA (*R* = 0.553, *P* < 0.001) and central subfield thickness (*R* = 0.408, *P* < 0.001) but not with DR severity. These data suggest that *reflectance-decorrelated foci* on OCTA images are clinically relevant as well as shed lights on the properties in diabetic hyperreflective foci.

## Introduction

Diabetic retinopathy (DR) often causes severe visual loss mediated via angiogenesis and the breakdown of the blood-retinal barrier (BRB)^1–3^. Vascular hyperpermeability promotes retinal thickening and the deposition of hard exudates, which result in visual impairment in DR. The central subfield (CSF) thickness is modestly related to the reduction of visual acuity (VA), and the subfoveal hard exudates lead to severe visual impairment in diabetic macular edema (DME)^4,5^.

According to pathohistological reports, hard exudates are the precipitates of extravasated components and contain hyaline, lipoproteins, or fat-filled phagocytes^6–8^. Hard exudates appear as confluent hyperreflective foci with various sizes, shapes, and reflectivity levels on spectral-domain optical coherence tomography (SD-OCT) images^9^. *In vivo*, high-resolution images obtained with adaptive optics scanning laser ophthalmoscopy (AO-SLO) have revealed round or irregular lesions within hard exudates, which had individual turnover rates^10^. Although these findings suggest heterogeneous components in hard exudates, it is unclear which *in vivo* imaging finding corresponds to cellular components and which finding corresponds to extravasated materials.

Recent advances in optical coherence tomography angiography (OCTA) have enabled noninvasive evaluation of retinal vasculature^11–13^. Differences in the reflectivity levels between sequential B-scan images can be enhanced by several image processing algorithms. The movement of erythrocytes with high reflectance allows for the delineation of physiological and pathological vasculature in the retina^14–22^. Other highly reflective components, e.g., the nerve fiber layer, retinal pigment epithelium, ellipsoid zone of photoreceptors, and hyperreflective foci, are delineated on structural OCT images, although it remains to be elucidated whether decorrelation signals are derived from such components^23^.

In the current study, we characterized the decorrelation signals of hyperreflective foci within the inner nuclear layer (INL) and Henle’s fiber layer (HFL) and evaluated the relationship between clinical parameters and quantified decorrelation signal levels within the HFL in DR.

## Results

### Decorrelation signals of hyperreflective foci in the HFL

**Figure 1:**
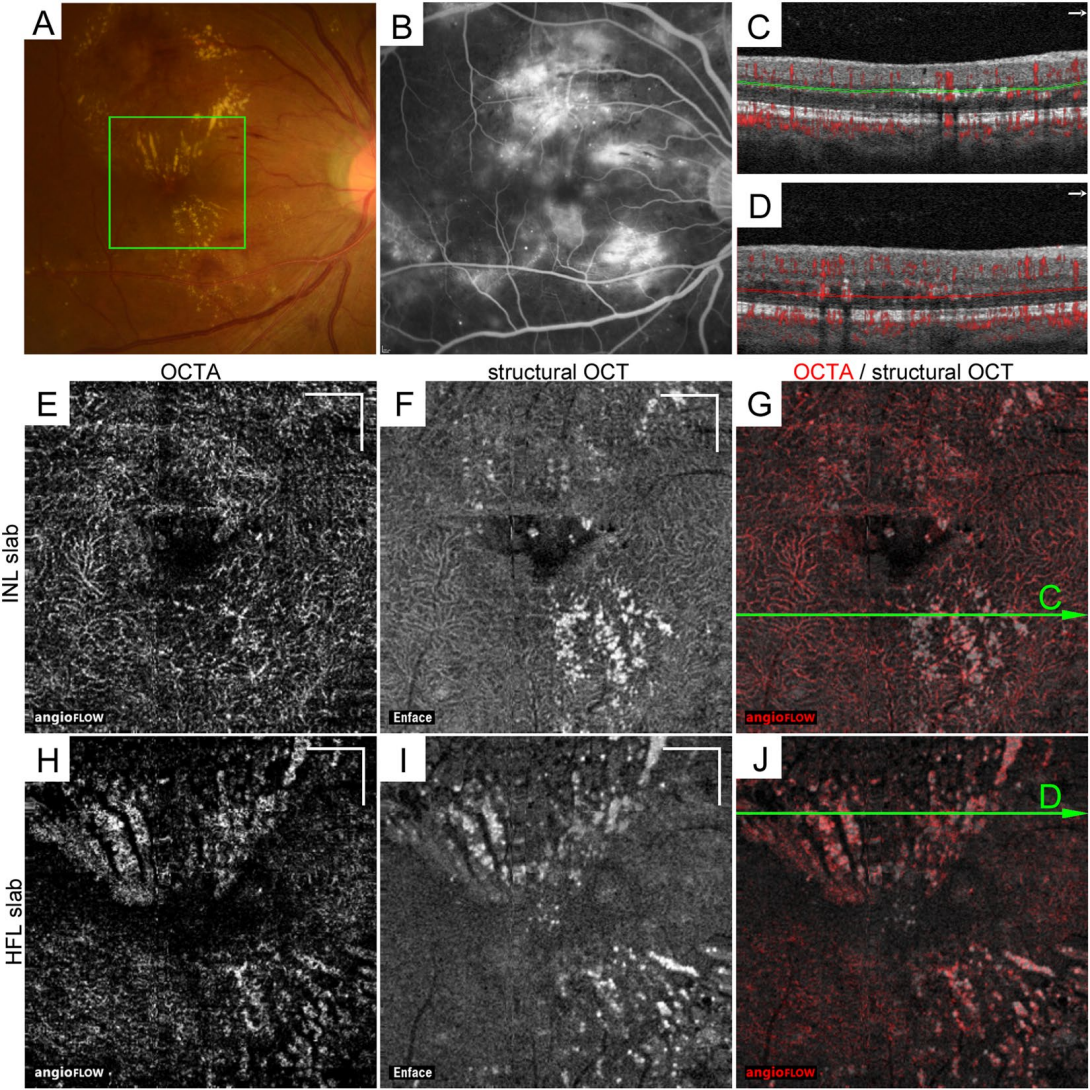
The distribution of hyperreflective foci on en-face OCT images in the INL and HFL in a 42-year-old patient with severe NPDR. (**A**) Hard exudates on fundus color photography. (**B**) The late-phase fluorescein angiography image shows hyperfluorescence in the macula. (**C**,**D**) The 10-μm-thick segmentation in the INL (**C**, between green lines) and HFL (**D**, between red lines) on B-scan images with decorrelation signals along the arrows in panels G and J, respectively. En-face OCTA (**E**,**H**) and structural OCT (**F**,**I**) and merged images (**G**,**J**; grayscale = structural OCT, red = OCTA) in the green rectangle in panel A. (**E**–**G**) The 10-µm-thick en-face image in the INL between the green lines in panel C. The structural OCT image delineates dot-like or spot-like hyperreflective foci. (**H**–**J**) The 10-µm-thick en-face image in the HFL between the red lines in panel D. The OCT image reveals that most hyperreflective foci are deposited in a radiating fashion and are partly colocalized to the decorrelation signal on the OCTA image. Scale bar = 500 μm.

In this study, we retrospectively reviewed the characteristics of hyperreflective foci on the structural OCT and OCTA images of 102 eyes of 66 patients with DR after the exclusion of 44 eyes without hyperreflective foci in the INL and HFL (Fig. 1). The patients’ characteristics are shown in the Table 1. Both sectional and en-face OCT images revealed hyperreflective foci in DR as previously described^9^. Confluent hyperreflective foci correspond to hard exudates in fundus findings and are mainly deposited from the INL to the HFL. Three-dimensional OCT imaging demonstrated that hyperreflective foci were randomly deposited in the INL (Fig. 1E–G), whereas most lesions in the HFL appeared to be radiating toward the foveal center (Fig. 1H–J). Although hyperreflective foci represent extravasation of blood components, these lesions were also identified in 60 eyes without center-involved DME.

**Table 1:** Patient characteristics.

Characteristic	
Eyes/patients	102/66
Age (years)	64.1 ± 11.0
Men/women	50/16
Diabetes duration (years)	13.2 ± 7.7
Hemoglobin A1c (%)	7.82 ± 2.07
Systemic hypertension (present/absent)	36/30
Dyslipidemia (present/absent)	18/48
LogMAR VA	0.061 ± 0.211
DR severity grade	
mild NPDR	8 eyes
moderate NPDR	47 eyes
severe NPDR	23 eyes
PDR	24 eyes
Center-involved DME	42 eyes
Ischemic maculopathy	—
CSF thickness (µm)	320 ± 99
EZ disruption (present/absent)	72/30
SSI	68.0 ± 5.4

**Figure 2:**
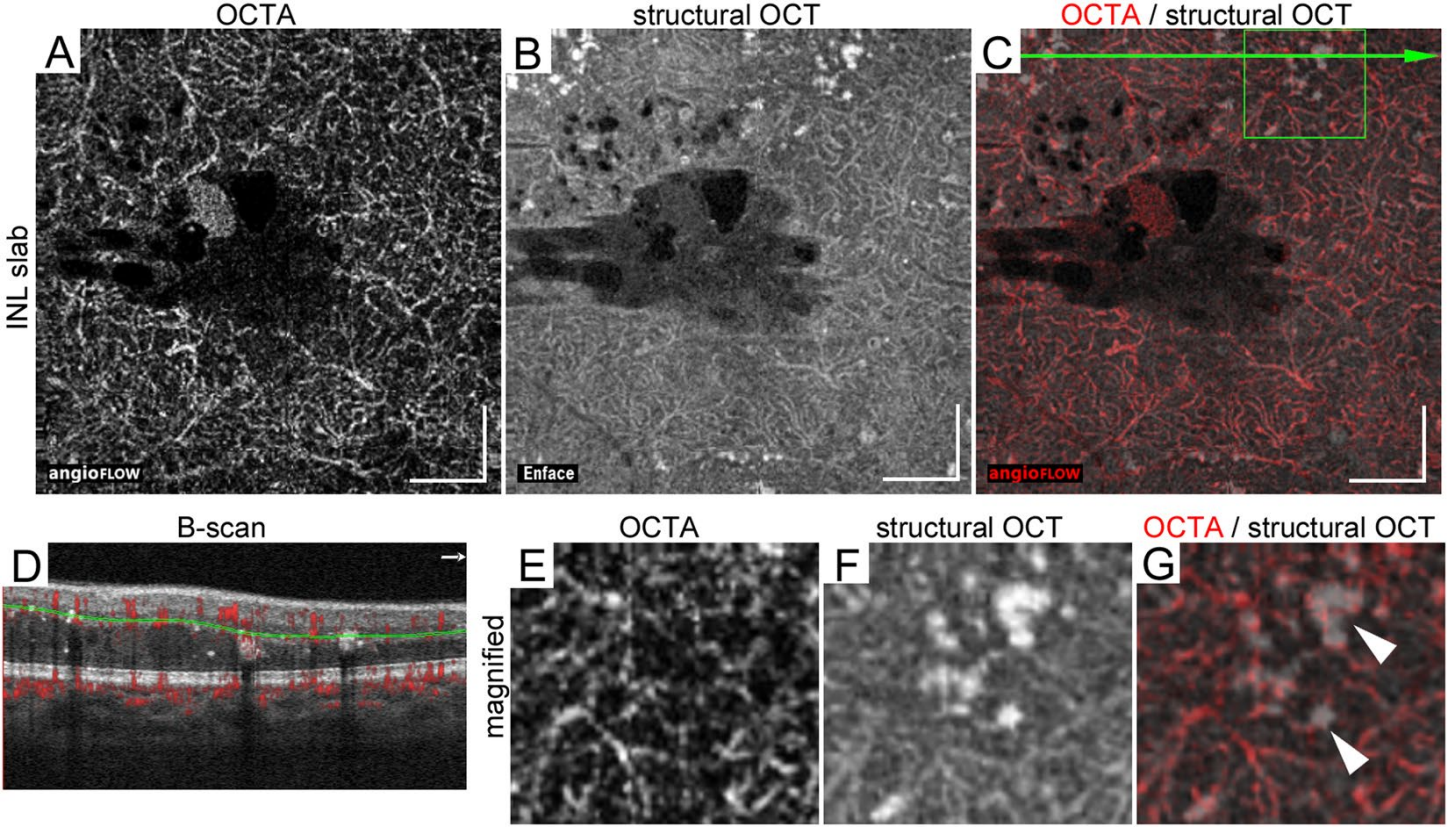
Hyperreflective foci attached to capillaries in the INL in a 67-year-old patient with moderate NPDR. The 10-μm-thick en-face OCTA (**A**), structural OCT (**B**), and merged images (**C**; grayscale = structural OCT, red = OCTA) within a central 3 × 3 mm square in the INL between the green lines in panel D. (**D**) The B-scan image with decorrelation signals along the green arrow in panel C. The magnified images of OCTA (**E**), structural OCT (**F**), and merged images (**G**) within the green square in panel C show the attachment of hyperreflective foci to the capillaries in this layer. Scale bar = 500 μm.

**Figure 3:**
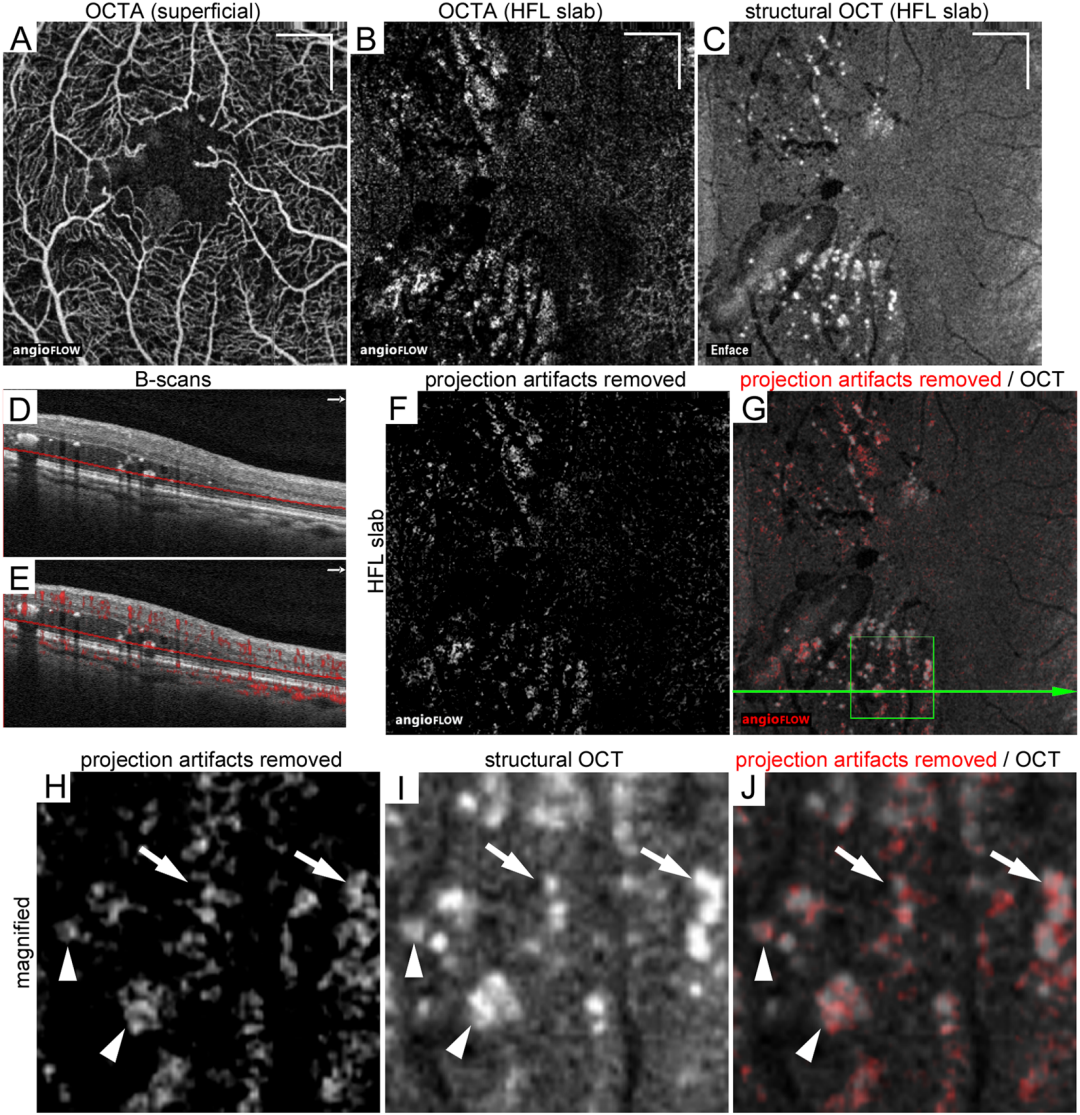
Reflectance-decorrelated foci correspond to parts of hyperreflective foci in the HFL in a 75-year-old patient with PDR. (**A**) En-face OCTA image in the superficial capillary layer. The 10-μm-thick en-face OCTA (**B**) and structural OCT (**C**) images. (**D**,**E**) The B-scan images without and with decorrelation signals along the green arrow in panel G. (**F**) Projection artifacts were removed from the OCTA slab in the HFL using the subtraction function in ImageJ. (**G**) The merged image of structural OCT and OCTA images after removal of projection artifacts. (**H**–**J**) The magnified images within the square in panel G. Decorrelation signals appear to be pseudopod-like or are wrapped around dot-like hyperreflective foci (arrows) and are partly colocalized to hyperreflective foci (arrowheads), referred to as *reflectance-decorrelated foci*. Scale bar = 500 μm.

**Figure 4:**
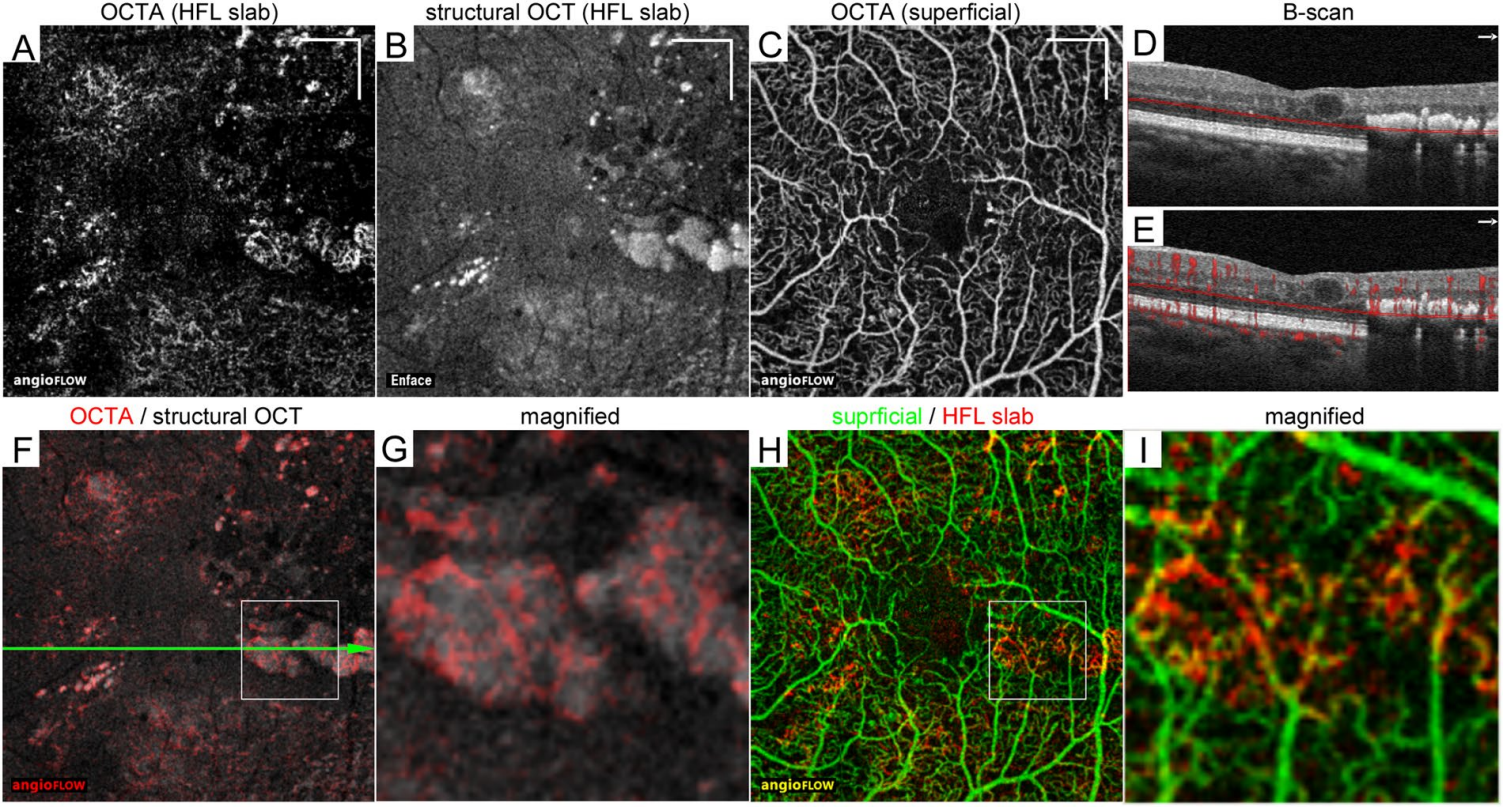
Projection artifacts in confluent hyperreflective foci within the HFL in a 60-year-old patient with moderate NPDR. The 10-μm-thick en-face OCTA (**A**) and structural OCT (**B**) images in the HFL between the red lines in panel D. (**C**) OCTA image in the superficial layer. (**D**,**E**) B-scan images without and with decorrelation signals along the green arrow in panel F. (**F**) The merged image (grayscale = structural OCT, red = OCTA) within a central 3 × 3 mm in the HFL. (**G**) The magnified image within the square in panel F. The decorrelation signal appears to be a meshwork-like capillary network. (**H**) The merged image of OCTA in the superficial layer (green) and HFL (red). (**I**) The magnified image within the square in panel H shows that the decorrelation signals in hyperreflective foci correspond to projection artifacts. Scale bar = 500 μm.

The comparative study between structural OCT and OCTA images revealed that decorrelation signals on OCTA images were rarely depicted in hyperreflective foci in the INL (Fig. 2). Hyperreflective foci in the INL were frequently attached to capillaries on OCTA images (Fig. 2E–G). By contrast, after the projection artifacts were removed, hyperreflective foci within HFL were often accompanied by decorrelation signals on OCTA images, referred to as ‘*reflectance-decorrelated foci*’ in this study (Fig. 3H–J). The well-circumscribed morphologies of decorrelation signals were sometimes matched to those of hyperreflective foci (Fig. 3H–J, arrowheads). Decorrelation signals other times appeared to be pseudopod-like or wrapping around dot-like hyperreflective foci, (Fig. 3H–J, arrows). The *reflectance-decorrelated foci* were also delineated around hyperreflective foci in some areas, e.g., spindle-shaped intraretinal spaces with mild reflectivity and the areas beneath hyperreflective foci. In some confluent hyperreflective foci, the decorrelation signals just corresponded to the projection artifacts derived from the inner retinal vasculature (Fig. 4H,I).

### Relation between clinical parameters and the decorrelation signal intensity of hyperreflective foci in the HFL

**Figure 5:**
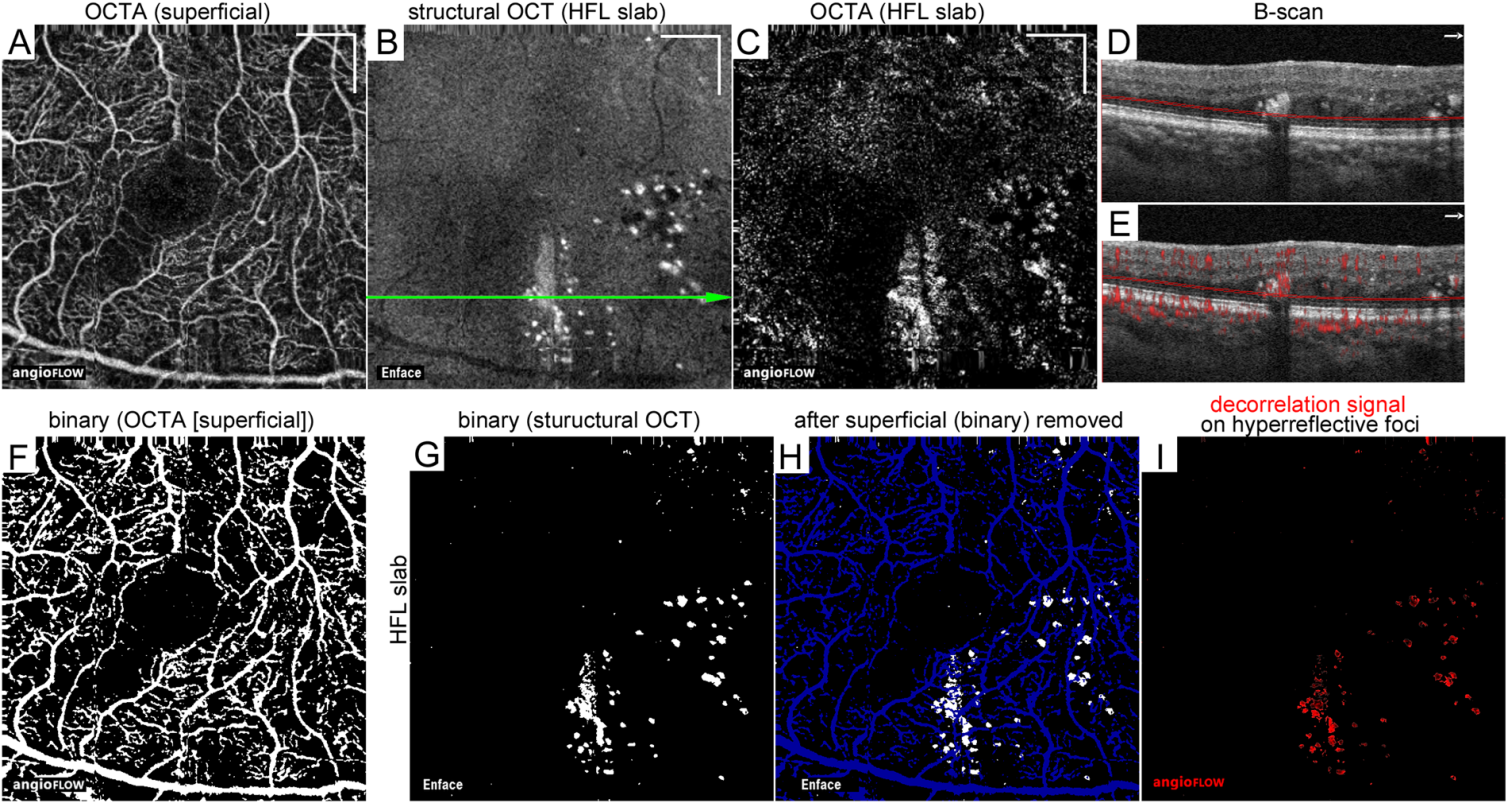
Quantification of the decorrelation signal intensity of hyperreflective foci in the HFL in a 63-year-old patient with moderate NPDR. (**A**) En-face OCTA image in the superficial layer. The 10-μm-thick structural OCT (**B**) and OCTA (**C**) slabs in the HFL between the red lines in panel D. (**D**,**E**) B-scan images without and with decorrelation signals along the green arrow in panel B. (**F**,**G**) Binary images of panels A and B, respectively, obtained using the global threshold function in ImageJ. (**H**) The areas of hyperreflective foci (white) after the exclusion of projection artifacts from the vessels in the superficial layer (blue). (**I**) The decorrelation signals (red) within highlighted areas in panel H. The signal levels in individual pixels were measured and averaged to calculate the decorrelation signal intensity. Scale bar = 500 μm.

**Figure 6:**
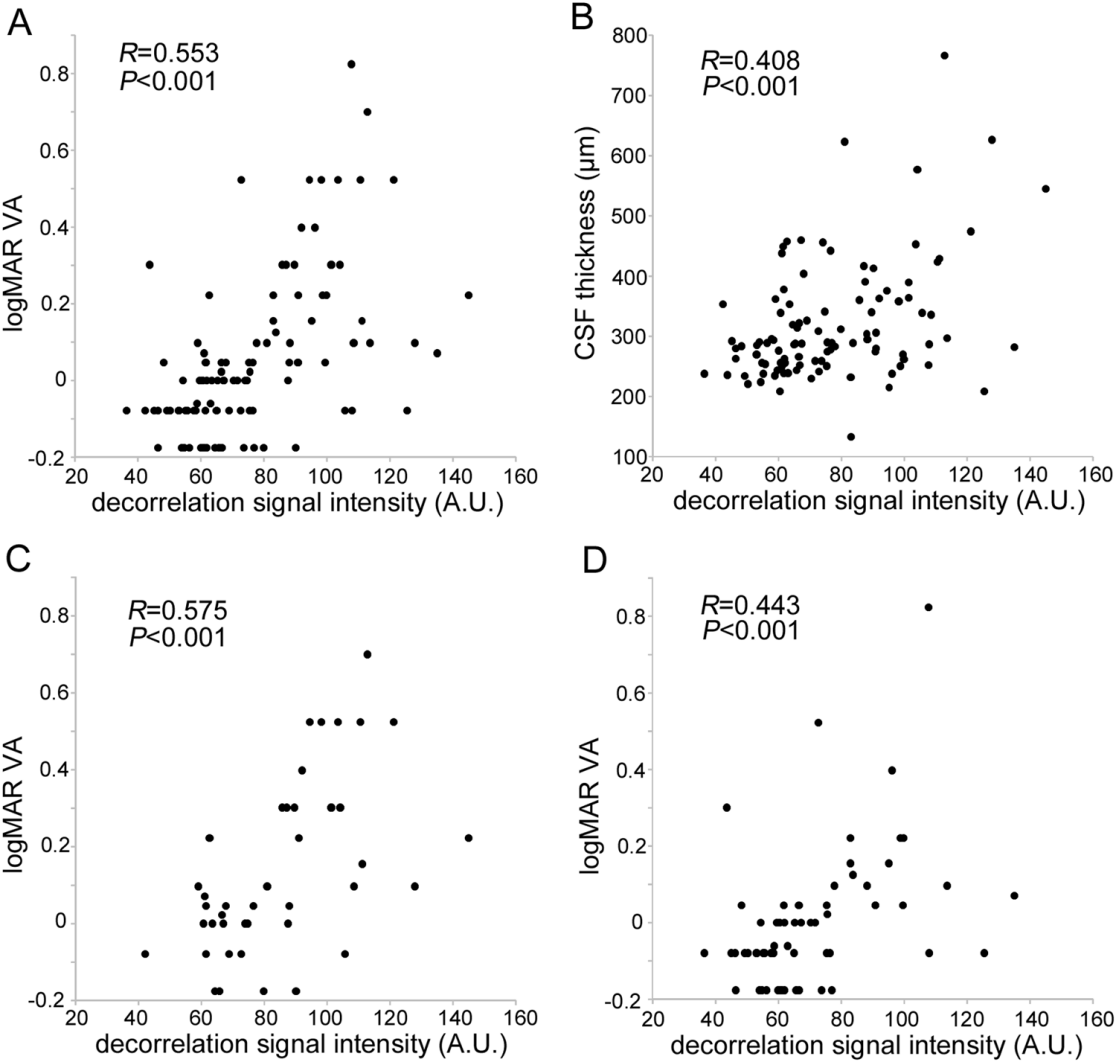
Association between logMAR VA and the decorrelation signal intensity of hyperreflective foci within the HFL. Correlation between logMAR VA and the decorrelation signal intensity of hyperreflective foci within the HFL in all 102 eyes (**A**), in 42 eyes with center-involved DME (**C**), and in 60 eyes without DME (**D**). (**B**) The association between CSF thickness and the decorrelation signal intensity.

In some eyes, most hyperreflective foci were accompanied by *reflectance-decorrelated foci*, and in other eyes, hyperreflective foci had a low decorrelation signal after the exclusion of projection artifacts. We thus quantified the decorrelation signal intensity of hyperreflective foci in the HFL and investigated its clinical relevance (Fig. 5). The preliminary comparison showed the good concordance between automatic detection and manual trace of the areas of hyperreflective foci in the HFL on en-face structural OCT images (intraclass correlation coefficient = 0.913 [95% confidence interval; 0.785–0.965]). Intriguingly, logarithm of the minimum angle of resolution visual acuity (logMAR VA) was modestly and positively correlated with the decorrelation signal intensity of the hyperreflective foci within the HFL in all 102 eyes (Fig. 6A). There was also an association between CSF thickness and the decorrelation signal intensity (Fig. 6B), which appears to be consistent with the correlation between logMAR VA and the decorrelation signal intensity in 42 eyes with center-involved DME (Fig. 6C). Furthermore, logMAR VA and the decorrelation signal intensity were positively correlated in 60 eyes without DME (Fig. 6D). Thirty-two eyes with not intact ellipsoid zone of photoreceptors (EZ) line at the fovea had greater signal intensity than 70 eyes with intact EZ line (88.3 ± 22.1 vs. 72.3 ± 20.6; *P* < 0.001). Multivariate analysis revealed that both the decorrelation signal intensity and the presence of EZ disruption but not CSF thickness were associated with logMAR VA in all 102 eyes (*β* = 0.409, *P* < 0.001, *β* = 0.358, *P* < 0.001, and *β* = 0.067, *P* = 0.448, respectively).

**Figure 7:**
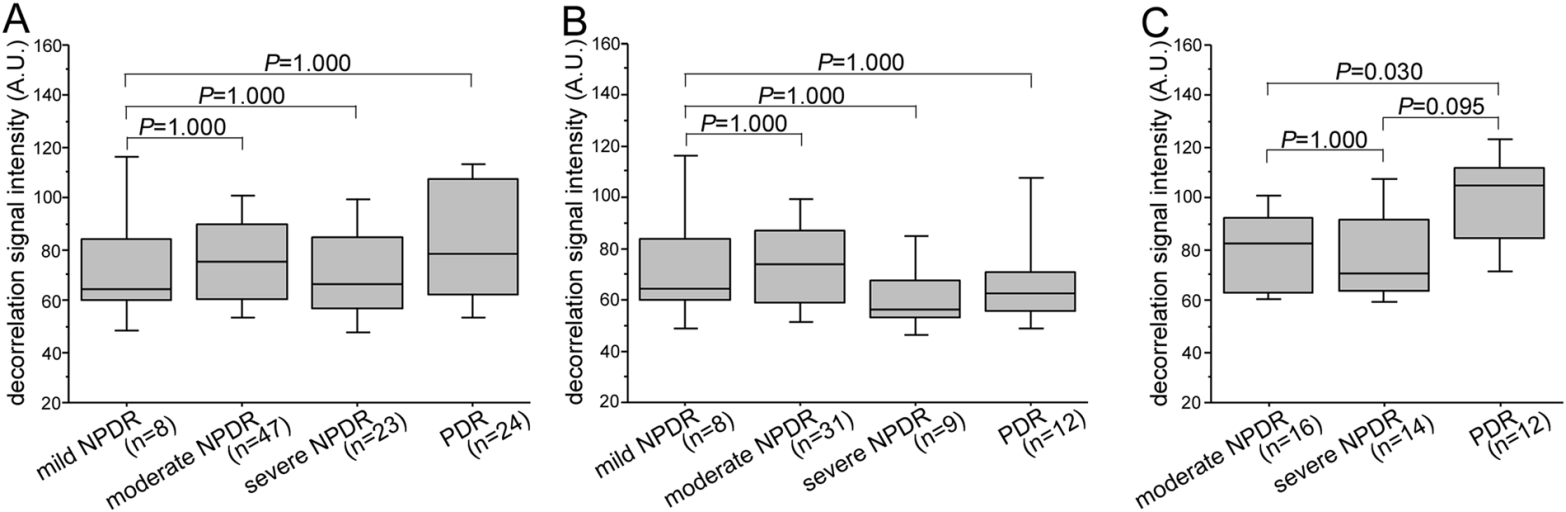
The decorrelation signal intensity of hyperreflective foci within the HFL in individual DR severity grades in all 102 eyes (**A**), 60 eyes without DME (**B**), and 42 eyes with DME (**C**).

The decorrelation signal intensity of hyperreflective foci in the HFL did not differ between individual grades of DR severity in all 102 eyes or 60 eyes without center-involved DME (Fig. 7A,B). In 40 eyes with DME, the signal intensity was higher in proliferative diabetic retinopathy (PDR) than in moderate nonproliferative diabetic retinopathy (NPDR) (Fig. 7C).

## Discussion

In the current study, we comparatively studied the structural OCT and OCTA findings of hyperreflective foci in DR. En-face images of the INL revealed randomly deposited hyperreflective foci that rarely had a decorrelation signal on OCTA images. By contrast, hyperreflective foci in the HFL were arrayed in a radiating fashion and were often accompanied by well-circumscribed lesions with a decorrelation signal, which were referred to as r*eflectance-decorrelated foci* as a novel OCTA finding. It suggests that hyperreflective foci or hard exudates are heterogeneous in shapes, positions, and optical properties. Intriguingly, statistical analysis demonstrated that the decorrelation signal intensity of the hyperreflective foci in the HFL was clinically relevant in DR. Further investigation should elucidate the effects of *reflectance-decorrelated foci* on neuroglial dysfunction in diabetic retinas.

Multivariate analysis revealed that visual impairment was associated with both foveal photoreceptor damage and the decorrelation signal intensity of hyperreflective foci in the HFL, rather than CSF thickness in DR. Generally, CSF thickness is modestly correlated with VA reduction in DME^4^, suggesting that factors other than the magnitude of edematous changes influence visual impairment. Since there was the correlation between CSF thickness and the decorrelation signal intensity in this study, we considered that vascular hyperpermeability regulates both *reflectance-decorrelated foci* and retinal edema and that *reflectance-decorrelated foci* are a novel factor affecting visual dysfunction. In addition, the decorrelation signal intensity was associated with morphological damage in foveal photoreceptors. These data might allow us to hypothesize that hyperreflective foci with decorrelation signals, which would correspond to parts of lipid-laden macrophages or deposited lipoproteins, contribute to foveal photoreceptor damage as well as other unknown neuroglial impairment. Further analyses regarding the local associations between the decorrelation signals and structural OCT would lead to better understanding of the pathogenesis in DR.

Several publications suggest the clinical relevance of hyperreflective foci in DR^24–28^. Hyperreflective foci in subretinal fluids can predict the development of subfoveal hard exudates and concomitant poor visual prognosis after intervention for DME^27^. In a cross-sectional study, hyperreflective foci in the outer retinal layer were associated with both photoreceptor damage and visual impairment in DME^26^. These publications are consistent with the association between visual reduction and the *reflectance-decorrelated foci* in the HFL in the current study. Hard exudates or hyperreflective foci represent either the dynamic extravasation or static deposition of blood components. It may allow us to hypothesize that the dynamic optical properties in reflectance-decorrelated foci represent the extravasation and concomitant neuroglial dysfunction rather than the deposited components.

We could not conclude the origin of decorrelation signals in hyperreflective foci; the movement of highly reflective materials or scattering fluctuation of reflective small particles, e.g., high-density, low-density lipoproteins, or microvesicles. A clinical publication documented that subretinal hyperreflective foci migrate toward the fovea during the absorption of subretinal fluids^29^. Intraretinal hyperreflective foci also increase in number and size during anti-VEGF treatment for DME, indicating the dynamic rearrangement of hyperreflective foci^30^. These publications suggest that these types of hyperreflective foci contain dynamic and hyperreflective materials at least in part and might correspond to lipid-laden macrophages migrating in intraretinal or subretinal spaces. It might agree with the dynamic optical properties in *reflectance-decorrelated foci* in the HFL. Compared with the flow of erythrocytes in blood vessels, the migration speed of phagocytes is generally slow in retinas^31^. Decorrelation signals might be dependent on morphological changes in microglia/macrophages at the same position or the intracellular rapid trafficking of organelles, e.g., endosomes and lysosomes, containing highly reflective materials^32,33^. *Reflectance-decorrelated foci* often appeared to be pseudopod-like or wrapping around dot-like hyperreflective foci and were not completely matched to hyperreflective foci. Hard exudates contain small reflective particles, e.g., lipoproteins, from which scattering fluctuation can be derived^34^. The future study has to explain the dynamic optical properties in parts of hyperreflective foci in DR.

Hyperreflective foci were attached to capillaries in the INL, and the decorrelation signals were not derived from themselves. Since hard exudates are often observed at the boundary of retinal edema, they might be deposited during the absorption of extracellular fluids^35^. In other words, as smaller molecules, e.g., water and ions, can be absorbed, macromolecules might be concentrated and statically deposited around capillaries. Another potential explanation is that static lipid-laden macrophages are attached to the vascular basement membrane mediated via adhesion molecules. Therefore, lipid-laden macrophages might reside around capillaries, similar to foam cells beneath the endothelial cells in atherosclerosis^36^.

This retrospective study has several limitations. We saw the segmentation errors in several cases, which might have influences on quantification of the decorrelation signal intensity and CSF thickness. The decorrelation signals were affected by projection artifacts, although we excluded areas with such artifacts in the quantitative analyses. Edematous changes or hyperreflective lesions, that is, shadow artifacts, in inner retinal layers reduce the reflective signals beneath them^37^. We could not completely exclude the possibility of a ‘false positive’ decorrelation signal, which is generally considered to be delineated around hyperreflective material. Since the optical axial resolution is 5 µm in this device, the thin slab images in the INL and HFL were approximately but not exactly created by the manufacturer’s software. Further advances in imaging acquisition and processing would improve the analyses and interpretation. We evaluated hyperreflective foci on en-face images using the manufacturer’s software, although three-dimensional analyses would provide a better understanding of such lesions. The current study documented hyperreflective foci in DR in a Japanese population in a single center. Further prospective studies should determine whether the data are generalizable to other chorioretinal diseases in multiple centers using other OCTA devices or other segmentation methods for hyperreflective foci.

In conclusion, for the first time, we documented the decorrelation signal derived from parts of hyperreflective foci within the HFL, referred to as *reflectance-decorrelated foci*, as a novel OCTA finding, and would promote the understanding of clinically relevant hyperreflective foci in diabetic retinas.

## Methods

### Participants

In this retrospective study, we reviewed 102 consecutive eyes of 66 patients with DR who visited the Department of Ophthalmology of Kyoto University Hospital from February 2015 to January 2017. DR eyes with sufficient quality OCTA images, which were acquired using an Optovue RTVue XR Avanti (Optovue, Fremont, CA), were consecutively included. An additional inclusion criterion was the presence of hyperreflective foci in both the INL slab (between 38 μm to 47 μm beneath the inner plexiform layer [IPL]/INL boundary) and HFL slab (between 91 μm to 100 μm above Bruch’s membrane) of structural OCT images within a central 3 × 3 mm square. The exclusion criteria were the presence of other chorioretinal diseases, glaucoma or ocular hypertension; an axial length shorter than 22.0 mm or longer than 26.0 mm; preretinal hemorrhages within a central 3 × 3 mm square on color fundus photography; a history of any intervention for macular lesions; intraocular surgery other than cataract extraction; cataract surgery within 6 months of study enrollment; and a signal strength index (SSI) score of 60 or less determined by the default setting of this instrument. Forty-two eyes had center-involved DME according to the manufacturer’s software^38^.

All research and measurements adhered to the tenets of the Declaration of Helsinki. The Kyoto University Graduate School and Faculty of Medicine Ethics Committee approved the study protocol. All participants provided written informed consent before inclusion in the study.

### Image acquisition

We performed a comprehensive ophthalmic examination, and best-corrected decimal VA was converted to logMAR VA. Axial length was measured using partial coherence interferometry (IOLMaster, Carl Zeiss Meditec AG). OCTA images in the 3 × 3 mm square centered on the fovea were obtained using an Optovue RTVue XR Avanti (Fig. 1). Given the high speed of 70,000 A-scans/second and a light source of approximately 840 nm, this imaging device depicts motion-dependent decorrelation signals using the split-spectrum amplitude-decorrelation angiography (SSADA) algorithm. Two consecutive B-scans (M-B frames) at a fixed position were obtained before the image acquisition at the next position, followed by the calculation of the decorrelation between these positions. As a result, the motion of highly reflective materials was detected, and sequential B-scans side-by-side allowed for the construction of three-dimensional motion contrast AngioFlow images.

### Quantification

We measured the mean retinal thickness within 1 mm circle at the fovea as CSF thickness according to the manufacturer’s software^38,39^. Foveal photoreceptor status was also evaluated and classified into two categories; intact or not; on the retinal sectional images dissecting the fovea according to the previous reports^23,40^.

Confluent hyperreflective foci were mainly delineated in the INL and HFL, and two retinal specialists determined the presence or absence of hyperreflective foci in these layers (kappa coefficient = 0.812 and 0.874 in the INL and HFL, respectively); when these specialists disagreed, a third specialist participated. The optical or digital axial resolution is 5 or 3 µm in this device, respectively. Segmentation processes in the manufacturer’s software allowed us to create approximate en-face images in the superficial layer (from the inner boundary 3 μm beneath the internal limiting membrane [ILM] to the outer boundary 15 μm beneath the inner plexiform layer [IPL]). We further prepared 10 µm-thick slab images at the center of the INL (from the inner boundary 38 μm beneath the IPL to the outer boundary 47 μm beneath the IPL) and HFL (from 91 to 100 μm anterior to the retinal pigment epithelium-Bruch’s membrane complex) using the default setting of the segmentation and following the manual setting of the thickness. We evaluated the qualitative relationship between hyperreflective foci on en-face structural OCT images and decorrelation signals on en-face OCTA images in the INL and HFL.

Specifically, we quantified the decorrelation signal intensity of hyperreflective foci in the HFL according to the following three steps; 1. to determine the areas of hyperreflective foci in the HFL (Fig. 5G), 2. to remove the areas with projection of superficial capillaries (Fig. 5H), 3. to quantify the decorrelation signal levels (Fig. 5I). We first created binary images of en-face structural OCT images in the HFL using the global threshold function of ImageJ (NIH, Bethesda, MD) to select the areas of hyperreflective foci (Fig. 5B,G). The shapes of hyperreflective foci in the HFL slab were various, and their reflectivity was much higher than the background in the whole areas. We thus selected the global thresholding, by which such lesions were automatically segmented according to the signal levels of whole pixels. We further quantified such areas manually using freehand selections tool of ImageJ and compared them to the automatically quantified areas in order to confirm the concordance between hyperreflective foci determined by these two methodologies.

As the second step, we removed the areas with projection of superficial vessels from the structural OCT image in the HFL slab. En-face OCTA images in the superficial layer were binarized using the global threshold function of ImageJ (Fig. 5A,F). We prepared two binary images, i.e., the superficial OCTA images after gradation inversion and the structural HFL image (Fig. 5G). The correlation highlighter function of WCIF ImageJ plugin software (http://www.uhnresearch.ca/facilities/wcif/imagej/) was applied to these images to determine the pixels of hyperreflective foci in the areas without the projection artifacts (Fig. 5H).

The third procedure was to apply the intensity correlation analysis function in WCIF ImageJ plugin software to this processed image and en-face OCTA images in the HFL (Fig. 5I), which enabled us to compare the grayscale levels of individual pixels in two corresponding images. It allowed us to count the pixels with individual grayscale levels within the areas of hyperreflective foci in the HFL slab. The mean value of decorrelation signal levels in the hyperreflective foci was calculated and referred to as the decorrelation signal intensity in this study.

### Statistics

The results are expressed as mean ± standard deviation (SD). The agreement of qualitative or quantitative evaluation of hyperreflective foci was confirmed using the kappa coefficient or intraclass correlation coefficient, respectively. The data were analyzed using analysis of variance (ANOVA) with Bonferroni correction to evaluate differences among groups. Pearson’s correlation coefficient was calculated to test the statistical correlation. We applied forced-entry multiple regression analysis (decorrelation signal intensity of the hyperreflective foci in HFL, the presence of not intact EZ line, and CSF thickness as independent variables; logMAR VA as a dependent variable) to adjust for a confounding factor. *P* < 0.05 was considered significant.
